# Effectiveness of Multicomponent Home-Based Rehabilitation in Elderly Patients after Hip Fracture Surgery: A Randomized Controlled Trial

**DOI:** 10.3390/jpm12040649

**Published:** 2022-04-18

**Authors:** Haneul Lee, Seon-Heui Lee

**Affiliations:** 1 Department of Physical Therapy, College of Health Science, Gachon University, Incheon 21936, Korea; leehaneul84@gachon.ac.kr; 2 Department of Nursing, College of Nursing, Gachon University, Incheon 21936, Korea

**Keywords:** hip fracture, multicomponent rehabilitation, home-based rehabilitation, home exercise

## Abstract

This randomized controlled study aimed to assess the clinical effectiveness of an 8-week personalized multicomponent home-based rehabilitation (MHR) program by comparing it with a home exercise program after discharge. Forty patients (≥60 years old) who underwent hip surgery were randomly assigned to multicomponent home-based rehabilitation (MHR) and home exercise groups. The MHR program included strength, endurance, balance, and breathing exercises; modifications to the home environment; education on assistive device use; pressure ulcer care; nutrition management; and motivational counseling. The MHR group received 24 visits from rehabilitation staff for 8 weeks (3 times a week), while the home exercise group received home exercises focusing on strengthening described in the leaflet. The rehabilitation staff prescribed the intensity of exercise at the first visit, and the home exercise group exercised without supervision after that for 8 weeks. Both groups received a 10-min phone call once a week for overall counseling to ensure high adherence to home exercises. Among the 40 participants, 29 (72.5%) completed the trial. The primary outcomes were balance and mobility. Balance was assessed using the functional reach test (FRT), and the timed up-and-go test (TUG) was used to assess balance and mobility. Data were analyzed using the intention-to-treat principle. The MHR group showed significant improvement compared to the home exercise group for FRT (mean difference (MD) 4.4 cm; 95% confidence interval (CI) 1.0 to 7.8) and TUG (MD: −4.2 s; 95% CI −8.0 to −0.3) after 8 weeks of intervention. Subjective pain and physical components of general health-related quality of life also improved significantly in the MHR group. No serious adverse events related to the interventions were observed. The eight-week of MHR program can effectively improve balance and mobility.

## 1. Introduction

Hip fractures are one of the most common causes of disability and require medical care, especially among older people. The incidence of hip fractures increases with age and nearly doubles after the age of 50 years [1,2]. By 2050, the annual number of hip fractures is expected to rise, with a huge increase in the global elderly population potentially leading to an increased global economic burden in some countries [3]. According to the World Health Organization, hip fractures are the most damaging among major musculoskeletal diseases [4]. Hip fractures limit the functional independence of individuals in their activities of daily living (ADL) and are associated with high morbidity and mortality [5]. The 1-year mortality rate after hip fracture is approximately 14–30%. Thus, the first year after a hip fracture is the most critical period [6].

Hip surgery is preferred over conservative treatment because it improves recovery and shortens hospital stay [7]. However, pain, decreased range of motion, and weak muscle strength after surgery can lead to poor balance and gait speed, which affect mobility [8]. Thus, it is important to regain functionality and mobility to restore patients’ independence in ADL after hip surgery.

Hip surgery can shorten the length of hospital stay and improve hip function in the elderly; however, long-term rehabilitation is required to restore functional independence [9]. Since patients seek shorter hospital stays due to a shortage of beds and increased medical expenses, there is growing interest in home-based rehabilitation for long-term rehabilitation after discharge [10]. Furthermore, home-based rehabilitation has a positive effect on patients’ physical and mental functions. Home-based rehabilitation after hip surgery through professional and systematic interventions should be considered [11,12,13]. Recovery rates for patients who received home-based rehabilitation were higher than those for patients who received usual care or hospital-based rehabilitation [14].

As home-based rehabilitation is feasible and safe, it has been institutionalized in most countries [15,16]. However, evidence on the effect of home-based rehabilitation on these populations in Asian countries is not yet been well-established. A recent systematic review of the effectiveness of multicomponent home-based rehabilitation claimed that only three out of twenty-two studies were from Asia (Taiwan); therefore, the results should be cautiously applied to countries in Asia [15]. In addition, a recent survey on patients undergoing hip fracture surgery in Korea emphasized the need for and awareness of a home-based rehabilitation program for elderly patients who underwent hip fracture surgery [4]. By synthesizing evidence [10,17,18], expert opinions, and patient value/preference based on a previous survey [4], the present study developed an eight-week structured, personalized, multicomponent home-based rehabilitation (MHR) program for elderly patients who underwent hip surgery within the UK Medical Research Council’s framework for complex interventions [19]. This study aimed to assess the clinical effectiveness of this 8-week personalized MHR program by comparing it with a home exercise program undertaken without supervision after discharge. We hypothesized that this MHR program would improve balance and gait function and benefit overall health more than unsupervised home exercise alone.

## 2. Materials and Methods

### 2.1. Study Design

This randomized controlled trial was conducted in accordance with the Consolidated Standards of Reporting Trial (CONSORT) recommendations [20] and registered on the cris.org website (KCT0005502). The intervention was reported based on the template for intervention description and replication (TIDieR) checklist and guide [21].

### 2.2. Ethical Approval

The study was approved by the Gachon University Institutional Review Board (96-201911-HR-209-02) and conducted in accordance with the Declaration of Helsinki. The participants were informed about the study’s aim and the measures undertaken to protect their privacy before the study was conducted. The participants who agreed to participate in the study signed an informed consent form.

### 2.3. Participants

A total of 48 participants were initially enrolled in this study between October 2020 and December 2021. The inclusion criteria were age 60 years or older; admission to a medical center for a traumatic, non-pathologic hip fracture; surgery to treat hip fracture within 16 weeks; and ambulation without human assistance at the time of hip fracture. Participants were excluded based on their inability to follow the rehabilitation procedures, such as those with neurological disorders who could not perform ADL before the fracture, at least mild cognitive impairment (Mini-Mental State Examination score < 24), or inability to communicate. Furthermore, these patients may have limited lung function or unstable cardiovascular conditions.

**Figure 1:**
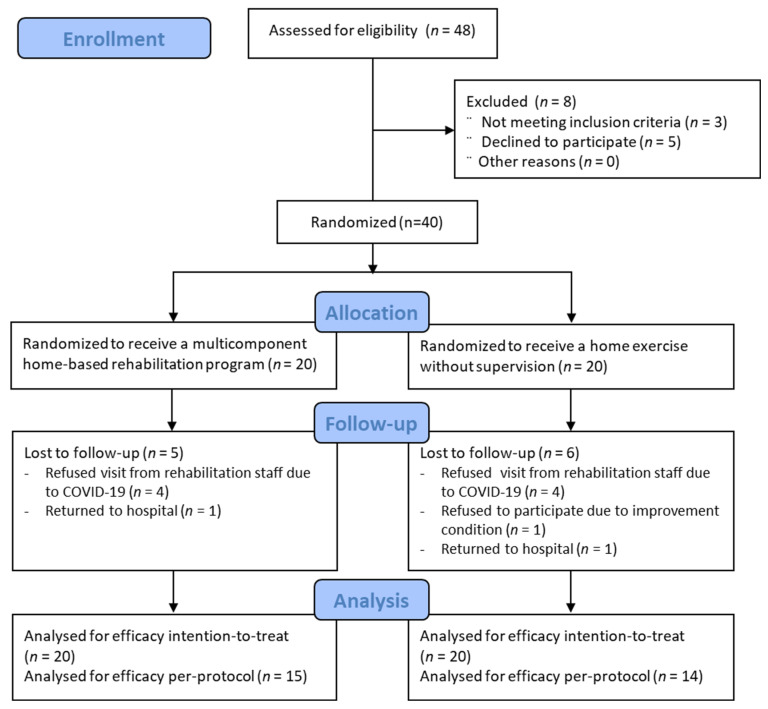
Flowchart of the study.

Of the 48 patients, five declined to participate in the study, and three did not meet the inclusion criteria. Finally, 40 patients (mean age 76.6 years, 75.0% women) were included in the study. These patients were randomly assigned to the MHR (*n* = 20) or home exercise (*n* = 20) groups (Figure 1).

### 2.4. Randomization and Blinding

Simple randomization was independently conducted by a study coordinator who was not involved in the assessment and data analysis processes, and concealed allocation was performed using a computer-generated randomized table of numbers before data collection. This ensured that all study investigators assessed the outcome variables and that the participants remained blinded to the allocation until the completion of the analysis. To reduce bias, the participants were informed about being randomly provided with either a specific or non-specific rehabilitative exercise intervention but not about the number of home visits.

### 2.5. Intervention

Physical therapists and visiting nurses provided 24 sessions of 60 min MHR interventions (three times a week for 8 weeks). At study initiation, physical therapists and nurses were randomly assigned to participants in the MHR group. Rehabilitation staff who provided MHR intervention to the participants completed 40 h of rehabilitation training developed by the research team for hip fracture patients.

The MHR provided personalized therapeutic exercise focusing on lower extremity strengthening, balance, and mobility function and was developed based on research evidence [4,10,17,18], expert opinion, and patient value/preference [4]. Strength exercises targeting the hip flexors, abductors, knee flexors, and extensors and intensity were decided depending on the patient’s strength using a microFET2 handheld dynamometer (Hogan Scientific, Salt Lake City, UT, USA). The participants also performed four tailored balance tasks using weight bearing: sit-to-stand, tapping the foot, stepping grid, and stepping up on a box [22,23]. Endurance exercises included indoor or outdoor walking; however, outdoor walking was performed only when possible. The target intensity was 50% of the age-predicted maximum heart rate or Borg rating of perceived exertion scale (range, 7–20), with a target intensity of 10–12 for participants on β-blocker medications. Breathing exercises and incentive spirometry training were also conducted [6,24]. The target intensity was decided on an individual basis by our rehabilitation staff and reassessed every two weeks. Moreover, since rehabilitation was conducted at home, modifications to the home environment, education on assistive device use, pressure ulcer care, nutrition management, and motivational counseling were included in the program for comprehensive rehabilitation.

The home exercise group was provided a rehabilitation leaflet for patients who underwent hip fracture surgery. The exercises included in the leaflet were lower-limb-strengthening exercises. During the initial assessment in the participants’ homes, a physical therapist educated them on the exercises described in the leaflet. These exercises were divided into three phases (early, mid, and end phases), and the physical therapist prescribed the intensity of exercise using a microFET2 handheld dynamometer through re-evaluation at weeks 0 and 4. Modifications to the home environment were assessed only at the first visit. In addition, the leaflet included assistive device use, pressure ulcer care, and nutritional management.

Both groups received a 10 min phone call with general medical questions, nutritional counseling, and encouragement to discuss their rehabilitation program or home exercise once a week for overall counseling to ensure high adherence to home exercise. Moreover, caregivers were instructed on how to manage patients with hip fractures at the first visit.

Visit adherence was defined as the number of completed therapist visits divided by the number of expected visits (24 sessions) within eight weeks.

### 2.6. Outcome Measures

The primary outcomes in this study were changes in balance and mobility over eight weeks, measured using the functional reach test (FRT) and timed up-and-go test (TUG). The FRT is a reliable assessment of balance in older adults after hip fracture [25]. The participants were asked to stand and extend their arms forward as far as possible with 90° shoulder flexion while remaining in contact with the ground [26,27]. The distance between the fingertip starting point and endpoint was measured [27]. The TUG test has been validated to assess balance and functional mobility [28,29]. Each participant was required to stand up from a standard armchair, walk forward 3 m, turn, walk back to the chair, and sit down [30,31]. The participants who required 30 s or less to complete this task tended to be more independent in their ADL [28,31].

The secondary outcomes included muscle strength, pain, ADL, quality of life (QoL), balance confidence, and depression. Muscle strength of the hip flexor, hip abductor, knee flexor, knee extensor, and grip strength was measured [32]. The amount of force generated by the hip flexor, abductor, and knee extensor muscles was measured using a microFET2 handheld dynamometer, and grip strength was measured using a Smedley-type dynamometer (Fabrication Enterprises Inc., Elmsford, NY, USA) [33]. Subjective pain was measured using a numeric rating scale (NRS), which has been validated for measuring postoperative pain intensity [34]. The 11-point numeric scale ranges from “0” (no pain) to “10” (worst pain imaginable) [35]. Participants responded to the following instruction: “Consider the amount of pain that you have experienced due to hip fracture surgery in your operated hip over the past 24 h”. The Korean version of the modified Barthel index (MBI) was used to evaluate ADL. It consists of 10 items describing ADL, and mobility is scored to measure the degree of assistance required by an individual [36]. Based on the degree of dependence, ratings were provided on a 5-point Likert scale, with a total score of 100. The higher the total score, the more independent the performed ADLs. The Barthel index has been validated for measuring functional recovery after arthroplasty for femoral neck fractures [37]. The Korean version of the Fall Efficacy Scale (FES) was used to measure balance confidence [38]. This is a 10-item questionnaire with a 10-point scale that ranges from “1” for “extreme confidence” to “10” for “no confidence at all” [39,40]. High scores indicated that the participants avoided activities because of fear of falling [39]. General health-related QoL was measured using the Korean version of the 36-item Short-Form Survey (SF-36) [41]. It consists of 36 items with eight domains of patient-reported surveys of patient general health [42]. SF-36 is scored as two summary measures: physical component summary (PCS) and mental component summary (MCS) [43]. In both measures, a lower score indicated “poor” health, while a higher score indicated “excellent” health [43].

Depression was assessed using the Korean version of the Center for Epidemiological Studies Depression (CES-D) scale (range 0–60) [44]. It is a self-reported scale that measures depressive symptoms in the general population (a higher score indicates more depressive symptoms) [45].

All assessments were performed at home by two experienced physical therapists at baseline (week 0), week 4, and week 8.

### 2.7. Sample Size

The sample size was calculated using G Power 3.1.9.7 software (Heinrich Heine University, Dusseldorf, Dusseldorf, Germany). The previous study showed the medium effect of TUG (*d* = 0.512) between the MHR and control groups [46]. However, no previous study reported effect size for time × group interaction in FRT or TUG. Therefore, the medium effect size of f = 0.25 was considered to calculate the sample size [47]. Twenty-eight participants were required to detect statistical significance when a clinically significant interaction was observed between the time points and groups, with effect size of f = 0.25, a significance level of 0.05, and a power of 0.80. An additional 30% of patients were recruited to provide unanticipated attrition.

### 2.8. Statistical Analysis

Analyses were performed based on an intent-to-treat principle using the IBM SPSS software (version 26.0; IBM, Armonk, NY, USA). The last observation carried forward method was used for participants who had completed at least one set of data measures. Data were summarized as means and standard deviations (SDs) for continuous variables and frequencies and percentages for categorical variables. The normality of the distributions was tested using the Shapiro–Wilk test. The Pearson chi-square test and independent *t*-test were performed for categorical variables and continuous variables, respectively, to compare the general characteristics of the participants and baseline data between the groups.

Mixed-effects linear regression models for longitudinal, repeated-measures data were used to examine changes in outcomes from baseline to weeks 4 and 8. These models included interaction terms between groups (intervention vs. control) and time (baseline, week 4, and week 8) that showed differential changes from baseline to week 4 and from baseline to week 8 between the intervention and control groups. The level of statistical significance was set at α = 0.05.

## 3. Results

Of the 40 study participants, 11 participants (five in the experimental and six in the control group) (27.5%) opted out of the study. The reasons for dropout were eight participants refusing visits from rehabilitation staff due to coronavirus disease 2019 (COVID-19), two returning to the hospital due to pain, and one participant’s condition improving faster than expected. Therefore, 29 patients completed the study. There were no significant differences in baseline function and general characteristics between participants who dropped out and those who completed the study. Visit adherence in the MHR group was 85.8 ± 23.3% for all participants, including dropouts, and 96.9 ± 5.7% for participants who completed the protocol.

The baseline characteristics were comparable between the groups except for the CES-D score (Table 1). Of the 40 patients, 30 (75.0%) were women; the mean BMI was 22.1 kg/m^2^, and the mean time since surgery was 59.7 days. The symptoms were mild to moderate in severity, and the mean baseline NRS pain score was 4.7. No serious adverse events related to the interventions were observed. All participants were able to complete the FRT and TUG tests at baseline. The baseline characteristics of the study participants are presented in Table 1.

The MHR group showed a significant improvement relative to the home exercise group in terms of the primary outcomes (Table 2). The between-group difference for the mean change from baseline in FRT was 2.4 cm at week 4 (95% CI 0.9 to 3.9) and 4.4 cm at week 8 (95% CI 1.0 to 7.8). For TUG, there was no significant difference in the mean changes from baseline between the groups at week 4 (−2.8; 95% CI −5.9 to 0.3), but the difference was significant between the groups at week 8 (−4.2; 95% CI −8.0 to −0.3). The changes between the group at week 8 can be considered as minimal clinically important difference (MCID) for older adults with hip fracture [48]. Mixed-effects models for repeated-measures analyses indicated that a significant difference in balance and mobility functions between the groups persisted at the week 8 assessment as well as when the trend for the change over time was modeled across the three time points (baseline, week 4, and week 8; Table 2).

With respect to secondary outcomes, subjective pain (−0.9; 95% CI −1.7 to −0.03) was significantly different between the MHR and home exercise groups in the mean change from baseline at week 8. Furthermore, there was a significant difference in subjective pain between the groups when the trend changed over time (*p* = 0.050). Regarding the general health-related QoL, the PCS score was significantly different in mean change from baseline to week 4 (4.5; 95% CI 0.0 to 9.1) and week 8 (7.8; 95% CI 1.5 to 14.1) between the groups. However, no significant difference was found in MCS scores at weeks 4 and 8. The home exercise group showed a significant effect on PCS eight weeks after the intervention (−5.3; 95% CI −8.9 to −1.6). However, this effect was not confirmed at week 4 (−2.7; 95% CI −5.8 to 0.3). Moreover, the change in PCS score over time was greater in the MHR group than in the home exercise group (*p* = 0.017). The between-group difference for the mean change from baseline in depression was −11.4 at week 4 (95% CI −18.5 to −4.4) and −13.4 at week 8 (95% CI −20.7 to −6.1). The change in depression over time was better in the MHR group (*p* = 0.001). However, after adjusting for baseline, as the baseline score was significantly different between the groups, the change was not significantly different between the groups (*p* = 0.201).

Knee flexor strength was significantly improved in the MHR group compared to that in the home exercise group at week 8 (11.5; 95% CI 0.8 to 23.7), but the group difference over time was not significant (Table 3).

For the primary outcomes, the results for the patients who completed the study were similar to the full cohort. Per-protocol analysis is described in Supplementary Table S1.

## 4. Discussion

This study found that the eight-week MHR program was effective in improving balance and mobility functions in elderly patients who underwent hip surgery, with the MHR group showing improvements in FRT and TUG compared to the home exercise group. After 4 weeks of intervention, 2.4 cm of statistically significant difference in improvement between the group was found in FRT, but the difference was not sufficient to be clinically significant, and no statistical and clinical difference between the group was found in TUG. However, the between-group differences of 4.4 cm for FRT and 4.2 s in TUG after 8 weeks of intervention were statistically and clinically significant. Our findings are consistent with those of previous research, which demonstrated a higher degree of recovery in balance function after home-based rehabilitation programs [46]. Similarly, a previous study reported no significant difference in TUG between the home-based rehabilitation program and the inpatient rehabilitation group [49]. Our findings suggest that MHR program should be applied for at least 8 weeks to improve balance and mobility function following hip fracture surgery.

Moreover, subjective pain was significantly less in the intervention group compared to that in the controls, with a between-group difference of 0.9 points. However, we found no evidence of an effect on self-reported function or muscle strength of the lower limb.

Both groups showed clinically significant improvements from baseline to week 4 and from baseline to week 8 for balance confidence (K-FES) and muscle strength but not for knee extensors and grip strength. Changes in outcome variables may reflect natural recovery after a hip fracture. However, changes in the usual care of patients with hip fractures, which were used as controls in other clinical trials [46,50], were smaller than those observed in the current study, suggesting that both the MHR program and home exercises provided greater benefits than usual care after hip fracture surgery in elderly patients. Although home exercises without supervision included only strengthening exercises of the lower limbs, the weekly motivational phone call for participants in this group may have had an effect beyond attention. This could explain why we could not find any significant difference in other variables between the groups although both groups experienced a significant increase in recovery after eight weeks of the intervention.

As the home exercise program focused on muscle strength of the lower limbs, both groups showed increased muscle strength. Rehabilitation is encouraged to reduce systemic complications or mortality caused by long-term immobilization after hip surgery, which is the main goal in the recovery process of patients with hip fracture patients [51]. In this respect, the lack of significant differences in muscle strength improvement between the two groups emphasizes the importance of continuing rehabilitation after discharge, suggesting that both interventions are effective in improving muscle strength. However, the MHR was superior to home exercises in terms of balance and gait function. Since balance is a complex skill that is achieved through the integration and coordination of the musculoskeletal and neural systems, it would have been improved in the MHR group that received training for four personalized tailored balance tasks from the rehabilitation staff. Considering that both muscle strength and balance ability are key factors in fall prevention among the elderly, the MHR program used in this study is meaningful.

Regarding self-rated general health-related QoL (SF-36), physical subdomains improved significantly in the MHR group compared to those in the home exercise group, whereas no effect on the self-rated mental health subdomain was found. This finding is slightly different from those of previous studies, in which both physical and mental subdomains improved or one of them changed after the intervention. This may be because home exercise alone was insufficient to generate a significant favorable effect on the subjective self-rated health status of the physical components. Furthermore, the present sample was susceptible to the influence of psychosocial and environmental factors as well as physical abilities. Regarding the mental component, there were significant differences before and after the intervention in both groups. However, the baseline score was an average of approximately 10 points higher than the physical component score, which made it difficult to obtain statistical significance. Thus, there were no significant differences between the groups.

ADL significantly improved from the baseline to week 8. However, no significant improvement was found at week 4 in the home exercise group, whereas the MHR group exhibited a change at weeks 4 and 8. Furthermore, no group differences were found at weeks 4 or 8. This may be a result of the home exercises’ focus on muscle strengthening, which took more time to improve ADL than the MHR program. Although the MHR program in the current trial mainly focused on strengthening, balance, and mobility training but not on ADL, since mobility and balance functions are included in MBI items, it seems that ADL improvement in the MHR group appeared earlier.

Furthermore, we found no evidence of any effect on depression. After adjusting for baseline data on depression, the depression score was not significantly different between the two groups, and no significant improvement after the intervention was found in either group. Similarly, a previous study comparing MHR and usual care reported no significant differences in CES-D scores between the groups, and no significant changes over time were found in the MHR group, with 20% of patients having a CES-D score of 16 or more after a month and 17% after six months [46]. Although the intervention group showed an average decrease of 17.5 points at week 8, indicating a large difference from the control group, with an average decrease of 4.1 points, statistically insignificant results appeared to have been obtained as a result of between-group differences in baseline depression. This may have occurred as a result of the rehabilitation staff providing face-to-face intervention during home visits as well as improvement in physical function [52].

One strength of this study was that the data were collected by blinded assessors. Furthermore, experienced physical therapists and nurses who had completed 40 h of rehabilitation training were used in the MHR program. This multi-professionally designed intervention strengthened nursing care through multicomponent home-based rehabilitation, including pressure ulcer care, nutrition management, and motivational counseling. Despite these strengths, the current study had several limitations. First, despite the fact that conducting assessments and interventions at participants’ homes eliminated important barriers to participation, the impact of COVID-19 caused more dropouts than expected. Although patients thought that home-visit rehabilitation reduced the risk of COVID-19 infection, elderly participants and their caregivers, in particular, showed fear of exposure to the virus through the rehabilitation staff. Second, follow-up assessments were not performed. To confirm the continuing effect of the intervention, further studies should include follow-up assessments at some point post-intervention. Third, home exercise adherence in the control group was not assessed. Although a phone call was made once a week to motivate the patients in the home exercise group to exercise, it was difficult to measure the exact effect of exercise without an exercise log.

Finally, although the study was equipped to detect clinically significant differences, the relatively small sample size, as compared to that in previous trials, was the biggest limitation of the current study. This is because home-based rehabilitation is not actively implemented in every country in the world [4]. As the MHR program was effective for elderly patients who underwent hip fracture surgery in the current trial, countries that have not yet implemented home-based rehabilitation should establish relevant policies to actively promote community healthcare to achieve continuous rehabilitation even after discharge.

## 5. Conclusions

Our results indicate that the eight-week MHR program, as compared to home exercise without supervision, resulted in a significant improvement in the balance function of elderly patients who underwent hip fracture surgery. Thus, the MHR program could be a feasible intervention for improving balance and mobility in patients. However, further research needs to be conducted with a longer duration and with a larger sample size to achieve more clinically significant results.

## Figures and Tables

**Table 1:** Baseline characteristics of the study participants.

	MHR Group (*n* = 20)	Home Exercise Group (*n* = 20)	*p*-Value
Age, years	78.9 ± 11.7	74.3 ± 9.2	0.174
Women, *n* *(%)*	15 (75.0)	15 (75.0)	
Height, cm	158.3 ± 9.3	155.2 ± 7.3	0.260
Weight, kg	55.4 ± 8.7	53.8 ± 10.3	0.612
BMI, kg/m^2^	22.0 ± 3.1	22.2 ± 3.4	0.926
Living status, *n* *(%)*			0.999
Living alone	2 (10.0)	2 (10.0)	
Living with family	18 (90.0)	18 (90.0)	
Time since surgery, days	55.0 ± 36.3	63.1 ± 26.2	0.439
Involved side, *n (%)*			0.337
Right	13 (65.0)	10 (50.0)	
Left	7 (35.0)	10 (50.0)	
Type of surgery, *n (%)*			0.109
Internal fixation	5 (25.0)	11 (55.0)	
Total hip arthroplasty	12 (60.0)	7 (35.0)	
Hemi arthroplasty	3 (15.0)	2 (10.0)	
Function Reach Test, cm	17.5 ± 8.1	18.9 ± 8.0	0.616
Timed Up-and-Go Test, sec	37.1 ± 10.5	34.3 ± 7.8	0.365
Pain Numeric Rating Scale (0–10)	4.9 ± 1.3	4.6 ± 1.5	0.551
Modified Barthel Index (0–100)	67.4 ± 23.6	78.7 ± 20.5	0.120
Fall Efficacy Scale (0–100)	53.4 ± 26.2	45.7 ± 18.6	0.304
Short Form 36			
Physical Component Score (0–100)	31.5 ± 9.7	37.3 ± 13.7	0.133
Mental Component Score (0–100)	40.6 ± 8.1	46.4 ± 14.4	0.123
Center for Epidemiological Studies Depression Scale (0–60)	28.7 ± 15.3	15.5 ±13.9	0.003
Muscle strength			
Hip flexor, kg	49.5 ± 15.2	51.1 ± 12.3	0.720
Hip abductor, kg	57.2 ± 16.3	59.2 ± 12.9	0.671
Knee flexor, kg	54.6 ± 16.9	60.5 ± 15.6	0.267
Knee extensor, kg	63.5 ± 15.8	70.6 ± 15.6	0.165
Grip strength, kg	16.6 ± 4.7	17.0 ± 4.2	0.770

Note: Unless otherwise indicated, all values are presented as mean ± SD.

**Table 2:** Primary outcomes for adjusted and unadjusted analyses between-group differences.

	Mean ± SD		Change from Baseline, Mean (95% CI)		Time × Group Interaction
	Week 4	Week 8	Week 4	Week 8	
**FRT (cm)**					
MHR group	21.4 ± 8.0	25.1 ± 9.6	−3.1 (−4.5 to −1.7)	−6.8 (−9.8 to −3.7)	
Home exercise group	21.1 ± 7.3	22.8 ± 7.7	−0.7 (−1.5 to −0.0)	−2.4 (−4.2 to −0.6)	
MHR vs. home exercise			2.4 (0.9 to 3.9)	4.4 (1.0 to 7.8)	0.015
**TUG (s)**					
MHR group	29.1 ± 10.9	24.8 ± 10.0	8.0 (5.7 to 10.3)	12.3 (8.9 to 15.7)	
Home exercise group	29.0 ± 9.1	26.6 ± 7.6	5.2 (3.0 to 7.5)	8.2 (5.8 to 10.5)	
MHR vs. home exercise			−2.8 (−5.9 to 0.3)	−4.2 (−8.0 to −0.3)	0.036

Abbreviations: FRT, functional reach test; TUG, Timed Up-and-Go Test.

**Table 3:** Secondary outcomes for adjusted and unadjusted analyses between-group differences.

	Mean ± SD		Change from Baseline, Mean (95% CI)		Time × Group Interaction
	Week 4	Week 8	Week 4	Week 8	
**Pain NRS (0–10)**					
MHR group	3.3 ± 1.5	2.4 ± 1.6	1.6 (1.0 to 2.2)	2.5 (1.8 to 3.1)	
Home exercise group	3.3 ± 1.4	3.1 ± 1.6	1.2 (0.6 to 1.8)	1.5 (0.8 to 2.1)	
MHR vs. home exercise			−0.3 (−1.1 to 0.6)	−0.9 (−1.7 to −0.0)	0.050
**K-MBI (0–100)**					
MHR group	73.9 ± 20.2	80.5 ± 17.8	−6.5 (−11.4 to −1.6)	−13.1 (−18.4 to −7.7)	
Home exercise group	80.8 ± 17.6	87.8 ± 14.9	−2.2 (−7.2 to 2.9)	−9.1 (−16.3 to −1.9)	
MHR vs. home exercise			4.3 (−2.5 to 11.1)	3.9 (−4.6 to 12.5)	0.425
**K-FES (0–100)**					
MHR group	41.6 ± 27.1	33.9 ± 26.5	10.3 (4.6 to 16.1)	17.0 (10.7 to 23.3)	
Home exercise group	35.3 ± 19.8	30.5 ± 15.1	10.2 (2.5 to 17.9)	14.9 (8.0 to 21.8)	
MHR vs. home exercise			−1.3 (−9.1 to 8.9)	−2.1 (−11.0 to 6.9)	0.893
**K-SF-36 (PCS) (0–100)**					
MHR group	38.7 ± 12.6	44.5 ± 16.9	−7.2 (−10.8 to −3.6)	−13.1 (−18.3 to −7.7)	
Home exercise group	40.0 ± 14.7	42.6 ± 14.4	−2.7 (−5.8 to 0.3)	−5.3 (−8.9 to −1.6)	
MHR vs. home exercise			4.5 (0.0 to 9.1)	7.8 (1.5 to 14.1)	0.017
**K-SF-36 (MCS) (0–100)**					
MHR group	49.6 ± 9.5	52.5 ± 10.4	−9.0 (−13.0 to −5.1)	−11.9 (−16.7 to −7.3)	
Home exercise group	52.3 ± 13.9	53.9 ± 12.9	−5.9 (−9.7 to −2.1)	−7.5 (−12.2 to −2.8)	
MHR vs. home exercise			3.1 (−2.2 to 8.5)	4.5 (−1.9 to 10.9)	0.242
**K-CES-D (0–60)**					
MHR group	16.1 ± 9.8	11.2 ± 7.7	12.6 (6.8 to 18.3)	17.5 (12.0 to 23.0)	
Home exercise group	12.9 ± 12.1	9.2 ± 8.7	1.1 (−3.4 to 5.6)	4.1 (−1.0 to 9.1)	
MHR vs. home exercise			−11.4 (−18.5 to −4.4)	−13.4 (−20.7 to −6.1)	0.001/0.201 ^†^
**Muscle strength**					
Hip flexor (kg)					
MHR group	61.3 ± 24.6	73.7 ± 25.1	−11.9 (−20.9 to −2.9)	−24.2 (−34.6 to −13.9)	
Home exercise group	59.9 ± 22.3	65.3 ± 21.2	−8.9 (−16.9 to −0.8)	−14.3 (−22.5 to −6.0)	
MHR vs. home exercise			3.0 (−8.8 to 14.7)	9.9 (−2.9 to 22.8)	0.183
Hip abductor (kg)					
MHR group	65.9 ± 22.0	76.4 ± 27.5	−8.7 (−15.2 to −2.1)	−19.2 (−27.9 to −10.6)	
Home exercise group	66.9 ± 19.1	71.6 ± 21.5	−7.8 (−14.9 to −0.6)	−12.4 (−21.3 to −3.6)	
MHR vs. home exercise			0.9 (−8.4 to 10.3)	6.8 (−5.2 to 18.8)	0.315
Knee flexor (kg)					
MHR group	70.7 ± 29.8	79.4 ± 30.2	−16.1 (−27.7 to −4.5)	−24.8 (−35.0 to −15.5)	
Home exercise group	69.7 ± 22.5	73..8 ±17.5	−9.2 (−17.2 to −1.3)	−13.3 (−20.6 to −6.1)	
MHR vs. home exercise			6.9 (−6.9 to 20.6)	11.5 (0.8 to 23.7)	0.167
Knee extensor (kg)					
MHR group	76.2 ± 29.5	91.2 ± 38.7	−12.7 (−22.9 to −2.6)	−27.7 (−14.5 to −13.9)	
Home exercise group	77.1 ± 22.0	85.1 ± 18.2	−6.5 (−14.1 to 1.0)	−14.6 (−23.4 to −5.8)	
MHR vs. home exercise			6.2 (−6.1 to 18.4)	13.1 (−2.8 to 29.1)	0.185
Grip strength (kg)					
MHR group	17.6 ± 5.7	19.1 ± 4.3	−0.9 (−3.0 to 1.1)	−2.5 (−4.2 to −0.8)	
Home exercise group	17.8 ± 6.5	18.5 ± 6.1	−0.8 (−2.8 to 1.3)	−1.6 (−4.3 to −1.2)	
MHR vs. home exercise			0.2 (−2.6 to 3.0)	0.9 (−2.2 to 4.0)	0.789

^†^ Adjusted for baseline. Abbreviations: NRS, numeric rating scale; K-MBI, Korean version of the Modified Barthel Index; K-FES, Korean version of the Fall Efficacy Scale; K-SF-36, Korean version of the 36-item Short-Form Survey; PCS, physical component score; MCS, mental component score; K-CES-D, Korean version of the Center for Epidemiological Studies Depression Scale.

## Data Availability

The datasets generated during this study are available from the corresponding author upon reasonable request.

## Supplementary Materials

The following supporting information can be downloaded at: https://www.mdpi.com/article/10.3390/jpm12040649/s1, Table S1: Outcomes for adjusted and unadjusted analyses between-group differences (per protocol).
