# Supplementary material for: Effectiveness of Multicomponent Home-Based Rehabilitation in Elderly Patients after Hip Fracture Surgery: A Randomized Controlled Trial
Source: J Pers Med. 2022 Apr 18;12(4):649. doi: 10.3390/jpm12040649 (PMC9027847; doi:10.3390/jpm12040649)
Supplement: Supplementary file 1 [file jpm-12-00649-s001.zip › jpm-1616916-supplementary.pdf]

Supplementary Table S1. Outcomes for adjusted and unadjusted analyses between−group differences (per protocol)

	**Mean ± SD**		**Change from baseline, Mean (95% CI)**		**Time × group interaction**
	**Week 4**	Week 8	Week 4	Week 8	
FRT, cm					
MHR group	21.6 ± 8.0	27.1 ± 9.6	−3.6 (−5.4 to −1.8)	−9.5 (−11.2 to −4.1)	
Home exercise group	19.5 ± 7.1	23.1 ± 8.2	−0.7 (−1.8 to 0.4)	−3.9 (−6.4 to −1.3)	
MHR vs. home exercise			2.9 (1.0 to 4.9)	5.8 (1.4 to 9.6)	0.044
TUG, sec					
MHR group	28.4 ± 11.4	25.9 ± 11.1	8.2 (5.3 to 11.1)	12.9 (8.3 to 18.8)	
Home exercise group	28.5 ± 8.2	26.3 ± 6.4	5.3 (2.7 to 7.9)	7.9 (5.1 to 11.1)	
MHR vs. home exercise			−2.9 (−6.6 to 0.8)	−4.7 (−10.6 to −1.9)	0.050
Pain NRS (0–10)					
MHR group	3.4 ± 1.4	2.2 ± 1.4	1.3 (0.6 to 2.0)	2.4 (1.6 to 3.2)	
Home exercise group	3.6 ± 1.2	3.3 ± 1.4	1.4 (0.6 to 2.1)	1.6 (0.9 to 2.4)	
MHR vs. home exercise			0.2 (−0.8 to 1.2)	−0.6 (−1.6 to 0.3)	0.150
K−MBI (0−100)					
MHR group	76.5 ± 20.7	82.2 ± 17.4	−6.1 (−10.5 to −1.6)	−11.8 (−18.4 to −5.2)	
Home exercise group	80.6 ± 15.1	88.5 ± 8.8	−3.5 (−10.4 to 3.4)	−10.2 (−18.6 to −2.8)	
MHR vs. home exercise			2.6 (−5.2 to 10.4)	1.4 (−6.3 to 10.5)	0.560
K−FES (0−100)					
MHR group	40.9 ± 27.0	33.3 ± 26.9	10.1 (3.2 to 16.4)	17.7 (11.1 to 24.3)	
Home exercise group	37.1 ± 17.6	30.3 ± 15.0	9.0 (4.5 to 13.5)	15.8 (11.0 to 20.7)	
MHR vs. home exercise			−1.1 (−8.8 to 6.7)	−1.9 (−10.1 to 6.3)	0.879
K−SF−36 (PCS) (0−100)					
MHR group	40.4 ± 13.8	46.3 ± 18.7	−6.9 (−11.9 to −1.8)	−12.8 (−20.2 to −5.4)	
Home exercise group	39.3 ± 15.4	42.2 ± 15.0	−1.9 (−5.5 to 1.7)	−4.8 (−8.9 to −0.7)	
MHR vs. home exercise			5.0 (−0.9 to 10.9)	8.0 (0.1 to 16.1)	0.072
K−SF−36 (MCS) (0−100)					
MHR group	50.2 ± 10.4	53.6 ± 11.1	−8.9 (−14.5 to −3.2)	−12.2 (−17.9 to −6.5)	
Home exercise group	53.0 ± 13.9	54.6 ± 12.2	−5.2 (−9.1 to −1.4)	−6.8 (−11.9 to −1.8)	
MHR vs. home exercise			3.6 (−2.9 to 10.1)	5.4 (−1.9 to 12.6)	0.237
K−CES−D (0–60)					
MHR group	15.9 ± 10.6	11.2 ± 8.5	12.3 (4.7 to 19.9)	17.0 (10.2 to 23.8)	
Home exercise group	11.4 ± 8.3	7.4 ± 5.0	1.8 (−4.2 to 7.7)	5.8 (−0.7 to 12.3)	
MHR vs. home exercise			−10.5 (−19.7 to −1.3)	−11.2 (−20.2 to −2.3)	0.009 / 0.953†
Muscle strength					
Hip flexor, kg					
MHR group	64.8 ± 23.8	80.3 ± 24.3	−13.7 (−22.8 to −4.)	−29.1 (−40.3 to −17.9)	
Home exercise group	59.2 ± 21.7	66.8 ± 20.8	−8.9 (−16.9 to −0.9)	−16.4 (−25.2 to −7.7)	
MHR vs. home exercise			4.7 (−6.8 to 16.3)	12.7 (−0.9 to 26.2)	0.096
Hip abductor, kg					
MHR group	69.4 ± 19.4	80.8 ± 22.6	−8.9 (−13.8 to −4.0)	−21.3 (−30.2 to −12.5)	
Home exercise group	65.7 ± 19.1	72.5 ± 16.6	−8.3 (−16.8 to 0.2)	−15.2 (−22.0 to −8.3)	
MHR vs. home exercise			0.6 (−8.6 to 9.7)	6.1 (−4.7 to 16.9)	0.340
Knee flexor, kg					
MHR group	71.5 ± 23.5	83.2 ± 30.2	−14.5 (−23.8 to −5.1)	−26.1 (−37.2 to −14.9)	
Home exercise group	68.8 ± 23.6	77.5 ± 16.6	−9.5 (−18.6 to −0.3)	−18.1 (−25.3 to −10.8)	
MHR vs. home exercise			5.0 (−7.5 to 17.5)	8.0 (−4.9 to 20.9)	0.428
Knee extensor, kg					
MHR group	74.1 ± 24.5	95.8 ± 32.0	−10.8 (−17.9 to −3.6)	−32.4 (−42.0 to −22.8)	
Home exercise group	78.2 ± 14.9	88.5 ± 13.5	−8.6 (−13.9 to −3.3)	−18.9 (−26.7 to −11.1)	
MHR vs. home exercise			2.2 (−6.4 to 10.8)	13.5 (1.5 to 25.4)	0.021
Grip strength, kg					
MHR group	17.5 ± 6.1	19.9 ± 4.8	−0.2 (−2.7 to 2.3)	−2.5 (−4.8 to −0.2)	
Home exercise group	16.7 ± 5.4	17.21± 6.3	−0.2 (−2.5 to 2.1)	−0.8 (−4.2to 2.5)	
MHR vs. home exercise			0.0 (−3.2 to 3.2)	1.7 (−2.1 to 5.5)	0.471

† Adjusted for baseline. Abbreviations: NRS, numeric rating scale; FRT, functional reach test; TUG, Timed Up and Go Test; K−MBI, Korean version of the Modified Barthel Index; K−FES, Korean version of the Fall Efficacy Scale; K−SF−36, Korean version of the 36−item Short Form Survey; PCS, physical component score; MCS, mental component score; K−CES−D, Korean version of the Center for Epidemiological Studies Depression Scale.
